# Biomimetic Mineralization on 3D Printed PLA Scaffolds: On the Response of Human Primary Osteoblasts Spheroids and In Vivo Implantation

**DOI:** 10.3390/polym13010074

**Published:** 2020-12-27

**Authors:** Marianna O. C. Maia-Pinto, Ana Carolina B. Brochado, Bruna Nunes Teixeira, Suelen C. Sartoretto, Marcelo J. Uzeda, Adriana T. N. N. Alves, Gutemberg G. Alves, Mônica D. Calasans-Maia, Rossana M. S. M. Thiré

**Affiliations:** 1 COPPE/Program of Metallurgical and Materials Engineering, Universidade Federal do Rio de Janeiro—UFRJ, Rio de Janeiro, RJ 21941-599, Brazil; marioliveira.bio@gmail.com (M.O.C.M.-P.); bnarj@metalmat.ufrj.br (B.N.T.); 2 Clinical Research Laboratory in Dentistry, Universidade Federal Fluminense, Rua Mario Santos Braga, 28/4° Andar, Niterói, RJ 24020-140, Brazil; 3 Post-Graduation Program in Science & Biotechnology, Universidade Federal Fluminense, Rua Mario Santos Braga, 28/4° Andar, Niterói, RJ 24220-140, Brazil; anacarol.batista.b@gmail.com (A.C.B.B.); gutopepe@yahoo.com.br (G.G.A.); 4 Clinical Research Unit, Antônio Pedro Hospital, Universidade Federal Fluminense, Rua Mario Santos Braga, 28/4° Andar, Niterói, RJ 24220-140, Brazil; 5 Oral Surgery Department, Dentistry School, Universidade Iguaçu, Avenida Abílio Augusto Távora, 2134, Nova Iguaçu, RJ 26260-045, Brazil; susartoretto@hotmail.com; 6 Oral Surgery Department, Dentistry School, Universidade Federal Fluminense, Rua Mario Santos Braga, 28/4° Andar, Niterói, RJ 24020-140, Brazil; mjuzeda@oi.com.br (M.J.U.); monicacalasansmaia@gmail.com (M.D.C.-M.); 7 Department of Oral Diagnosis, Dentistry School, Universidade Federal Fluminense, Rua Mario Santos Braga, 28/4° Andar, Niterói, RJ 24020-140, Brazil; adrianaterezinha@globo.com

**Keywords:** 3D printing, biomimetic, poly (lactic acid), spheroids, bone repair, 3D printed scaffold, bone morphogenetic protein 2, biomimetic apatite

## Abstract

This study aimed to assess the response of 3D printed polylactic acid (PLA) scaffolds biomimetically coated with apatite on human primary osteoblast (HOb) spheroids and evaluate the biological response to its association with Bone Morphogenetic Protein 2 (rhBMP-2) in rat calvaria. PLA scaffolds were produced via 3D printing, soaked in simulated body fluid (SBF) solution to promote apatite deposition, and characterized by physical-chemical, morphological, and mechanical properties. PLA-CaP scaffolds with interconnected porous and mechanical properties suitable for bone repairing were produced with reproducibility. The in vitro biological response was assessed with human primary osteoblast spheroids. Increased cell adhesion and the rise of in vitro release of growth factors (Platelet-Derived Growth Factor (PDGF), Basic Fibroblast Growth Factor (bFGF), Vascular Endothelial Growth Factor (VEGF) was observed for PLA-CaP scaffolds, when pre-treated with fetal bovine serum (FBS). This pre-treatment with FBS was done in a way to enhance the adsorption of serum proteins, increasing the number of bioactive sites on the surface of scaffolds, and to partially mimic in vivo interactions. The in vivo analysis was conducted through the implantation of 3D printed PLA scaffolds either alone, coated with apatite (PLA-CaP) or PLA-CaP loaded with rhBMP-2 on critical-sized defects (8 mm) of rat calvaria. PLA-CaP+rhBMP2 presented higher values of newly formed bone (NFB) than other groups at all in vivo experimental periods (*p* < 0.05), attaining 44.85% of NFB after six months. These findings indicated two new potential candidates as alternatives to autogenous bone grafts for long-term treatment: (i) 3D-printed PLA-CaP scaffold associated with spheroids, since it can reduce the time of repair in situ by expression of biomolecules and growth factors; and (ii) 3D-printed PLA-CaP functionalized rhBMP2 scaffold, a biocompatible, bioactive biomaterial, with osteoconductivity and osteoinductivity.

## 1. Introduction

Bone healing is a complex physiological process of reconstructing bone tissue that depends on the type and extent of the injury and patient’s age and gender [1,2]. The need for new materials to act as a tissue scaffold has increased considerably. The body can follow two routes for repair: healing and regeneration. Healing corresponds to fibrous accumulation in the injured area, with scar formation and loss of tissue function. Regeneration produces a new tissue without loss of function. In cases where the body cannot repair the injury by itself, some known clinical strategies are available, including the use of biomaterials, even though significant limitations affect the choice for treatment [3]. Autografts, where bone tissue is taken from the patient, allografts (bone harvested from cadavers), and xenografts (bone taken from another species) present potential risk of contamination, limited available quantities, and undesired immune response [4,5,6,7].

Tissue Engineering is conceptualized around a triad consisting of cells, regulatory signals, and scaffolds, promoting the regeneration of lost or damaged tissue. Researchers in the field of Bone Tissue Engineering (BTE) have faced the challenge of creating new alloplastic biomaterials capable of actin as three-dimensional (3D) scaffolds to enable cell migration, angiogenesis, new extracellular matrix deposition, mineralization, and regeneration of bone tissue [8,9,10].

The manufacture of tissue substitutes requires specific biomechanical and structural features. The choice of a biomaterial’s chemical nature depends on the substituted tissue, the purpose of its applicability, and the expected properties to achieve it [11]. Therefore, during its development, there is a need to investigate the surface chemistry available for cell attachment, the biology of the tissue that will come into direct contact with the implant, and the surface area, among other parameters. It must ensure that scaffolds will be seeded with suitable cells in vitro and then surgically implanted into the place of damage. Thus, research in Bone Tissue Engineering has challenged the creation of new porous 3D scaffolds that can mimic bone tissue with suitable physicochemical and biological features [12,13,14]. They must enable cell migration, angiogenesis, new extracellular matrix deposition, mineralization, and bone tissue regeneration. These scaffolds can be manufactured from synthetic or natural polymeric substrates, ceramics, or composites, using different fabrication approaches.

In this context, polymeric materials receive special attention in Tissue Engineering applications due to their controlled biodegradability and processability compared to other materials such as metals and ceramics. Biodegradable materials have excelled as scaffold candidates, allowing the grafted area’s gradual substitution with newly formed tissue. In this context, bioabsorbable polymers are great candidates for the fabrication of devices such as porous structures and temporary three-dimensional prostheses. It is expected that polymer implants will degrade by simple hydrolysis while providing support for cells to remodel the host tissue. Furthermore, its metabolites are incorporated into the biochemical pathways or excreted by the kidneys [15,16].

Aliphatic polyesters, such as polylactic acid (PLA), have stood out due to their biocompatibility, bioresorbability, and good thermal and physical properties in the production of sutures, manufactured orthopedic parts, microspheres for controlled release of drugs, and support for tissue regeneration [17]. PLA is approved by the FDA—Food and Drug Administration, for clinical trials and applications in contact with body fluids. PLA is obtained from lactic acid, an organic acid that originates from the fermentation of sugars obtained from renewable resources, such as sugarcane [18,19,20]. Another advantage of PLA is that it can be degraded by simple hydrolysis of its ester bonds, without enzymes or catalysts, and a second operation is not necessary for implant removal. PLA degradation results in lactic acid, which is usually present in the body [21]. Furthermore, there is a variety of possible techniques for PLA processing, such as injection molding, extrusion, film casting and electrospinning [22].

Additive Manufacturing (AM) technologies, also known as 3D printing technologies, enable the layer-by-layer, controlled production of porous customized scaffolds for BTE. Computer-aided design (CAD) files obtained from computer tomography, magnetic resonance imaging or scanner data can serve as templates for the fabrication of scaffolds, generating personalized devices for each patient [23,24,25,26,27,28,29]. Among AM technologies, Fused Deposition Modeling (FDM) is an easy and scalable processing technology that uses polymeric filament to create 3D geometries. The main advantages of FDM include: (i) no need for organic solvents, which tend to introduce some toxicity to the material, (ii) reproducibility and accuracy of produced parts, and (iii) a low cost in comparison with other 3D printing techniques [30,31]. This technology also allows obtaining scaffolds with interconnectivity and good porosity, which is necessary for new tissue ingrowth and nutrient and oxygen changes.

The surface characteristics of biomaterials are essential for successful medical applications, as they modulate the first contact with the biological medium and play an essential role in cell adhesion and proliferation [32,33]. It is known that calcium phosphate (CaP) coatings can increase synthetic scaffolds’ bioactivity [34]. The simulated body fluid (SBF) solution proposed by Kokubo in 1990 has been used as an effective method in the formation or precipitation of calcium phosphates (apatite) on the surfaces of different types of biomaterials, predicting their bioactivity in the bone tissue in vivo [35,36]. The biomineralization of bone scaffolds in SBF medium has become one of the most critical surface techniques by the deposition of apatite minerals that mimic bone tissue, consequently increasing their biocompatibility. One way to mineralize polymer’s surface is by direct contact with a solution rich in calcium and phosphate such as SBF, which has similar ionic concentration to that of the human extracellular fluid [37,38]. This fluid can be used to evaluate the bioactivity of artificial materials in vitro, and favor the apatite precipitation under biomimetic conditions, i.e., 37 °C and pH 7.4. The SBF treatment is independent of complex equipment or high temperatures and therefore represents a relatively simple technique to improve the biocompatibility and cell-surface interactions.

To assess the efficacy of surface functionalization in biocompatible biomaterials, the majority of in vitro assessments are performed through direct cell seeding, which creates a monolayer over the material surface [39,40]. However, new paradigms of in vitro studies in the 21st century include the use of microtissues and 3D culture models [41]. Compared to the 2D-monolayer model, the 3D cultured model can mimic the microenvironment in native tissue, with more precise tissue response simulation. Multicellular aggregates are formed by a process entitled self-assembly, when mono-dispersed cells attach through adhesion molecules, forming 3D microtissues called spheroids [42,43,44,45]. Tridimensional cell aggregates simulate gradients of oxygen, nutrients, metabolites, soluble signals, and cell-cell/cell-matrix interactions, which influence the physiological tissue responses [46,47]. The idea of assembling tissue spheroids into more complex structures has been suggested to produce improved scaffolds for tissue engineering [48] and as a predictive tool to investigate their biocompatibility with the target tissue [49].

CaP biomimetic coatings confer osteoconductivity to scaffolds, but not necessarily osteoinductivity property. Thus, one of the challenges in bone tissue engineering is the inclusion of biological mediators that contribute to improving tissue regeneration. Growth factors (GFs) can perform a range of regenerative functions and, among those, recombinant human bone morphogenetic protein-2 (rhBMP-2) is highlighted as one with the greatest osteoinductive potential. They are produced by osteoblasts and are associated with bone formation, recruiting osteoprogenitor cells [50]. Recombinant human BMP (rhBMP-2) is synthesized by genetic engineering and approved by the Food and Drug Administration (FDA) for applications in dentistry and orthopedic areas, usually incorporated into absorbable collagen sponges (ACS) for treatment of small defects [51]. However, the combination of rhBMP-2 and biomaterials may also represent a promising strategy for use in large defects.

The implantation of biomaterials, prostheses, or medical devices results in a homeostatic instability in the damaged region. Therefore, it is necessary to understand the material’s reactivity through in vitro assessments and animal models before clinical trials and application. In this context, the present study aimed to assess the effect of a biomimetic coating in 3D-printed PLA scaffolds through: (i) a tridimensional model of human primary osteoblast aggregates, assessing cell-surface interaction and the release of mediators of interest to bone tissue engineering and (ii) an in vivo animal model to investigate the osteogenic potential of these PLA-CaP scaffolds associated with rhBMP-2 for bone tissue engineering.

## 2. Materials and Methods

### 2.1. Scaffold Fabrication

**Figure 1:**
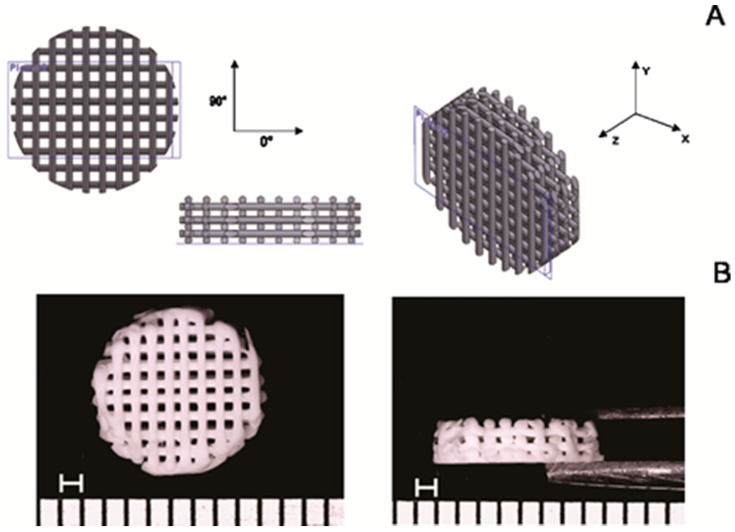
PLA scaffold design. The computer-aided design (CAD) model of scaffolds (**A**); Representative macrographs of printed PLA scaffolds (**B**) (Scale bar = 1 mm).

Poly (lactic acid) (PLA) was commercially obtained as a filament with 1.75 mm in diameter in white color, manufactured by Shenzhen eSun Industrial Co., Ltd. (lot 20150427-5, Nanshan District, Shenzhen, China), acquired from the reseller company Comercial Sazobras Ltd.a. (Campinas, São Paulo, Brazil). Cylindrical porous digital model with 8 mm in diameter per 2 mm in height (for application in rat’s calvaria), lattices with orthogonal beams with layer orientation of 0–90°, the distance between struts of 0.50 mm, and diameter of each strut of 0.30 mm was designed in SolidWorks^®^ 2014 software (Figure 1).

Parts were projected to create a pore size in the range of 500 µm, described as adequate for extracellular matrix deposition. The porosity was expected at around 50%. 3D Cloner FDM machine (Microbrás, Toledo, Paraná, Brazil) was used to printer the scaffolds, with the print head extrusion temperature set at 220 °C. Posteriorly, PLA scaffolds were subjected to the alkaline hydrolysis process (1M NaOH) at 65 °C for 30 min. After this step, PLA scaffolds were washed twice with deionized water to eliminate any residues of NaOH. This process allows the increasing of hydroxyl and carboxylic groups on the surface of scaffolds due to the breakage of the ester bond of the polymer chains. The carboxylic groups can serve as mineral nucleation sites due to their ability to chelate calcium ions from SBF solution. It is known that the alkaline treatment of polyesters increases the hydrophilicity of scaffolds, also increasing the roughness and the carboxylic acid density on the surface of polymer supports, which has a significant impact on cellular adhesion [52,53]. Then, the aim of scaffolds’ pre-treatment was to increase the number of active sites for cellular binding to materials, to favor apatite deposition by the biomimetic coating and to remove possible non-projected filaments present in the printed scaffolds.

### 2.2. Apatite Formation on the Surface of PLA Scaffolds (PLA-CaP)

According to the protocol established by Kokubo and Takadama (2006) [36], the SBF solution was prepared in 1.0 and 1.5 concentrations, by dissolving the following reagents: NaCl, NaHCO_3_, KCl, K_2_HPO_4_ 3H_2_O, CaCl_2_, Na_2_SO_4_ and MgCl_2_. 6H_2_O in distilled water. The pH was adjusted to 7.4 by adding 0.01 M Tris-hydroxymethyl aminomethane ((CH_2_OH)_3_CNH_2_) (Tris) and 0.01 molL^−1^ HCl at 36.5 °C. The ionic concentration of 1.0 and 1.5 SBF solutions in comparison with those of human blood plasma are show in Table 1.

Apatite formation on PLA scaffolds’ surface was submitted to a procedure inspired by Chim et al. [54]. A pilot was conducted to determine the scaffolds’ optimal exposure time in SBF solution at room temperature (data not shown). It was established that a total of 14 days of exposure were sufficient to form a homogeneous apatite layer since longer periods (21 days) produced an irregular and thick apatite layer with flaking points.

Scaffolds were immersed for seven days in 15 mL of SBF (pH 7.4, 37 °C) with ionic concentration similar to human blood plasma (1.0 SBF). In this period, it is expected initial mineral nucleation into carboxylic groups, promoting a good adhesion between the coating and the substrate [55,56]. After this, scaffolds were re-immersed in 1.5 SBF solutions for an additional 7 days, with the solution refreshed every two days. This step was given to making calcium phosphates crystals grow. On day 14, after the CaP coating (PLA-CaP), scaffolds were washed with deionized water and dried at room temperature. All 3D printed scaffolds were sterilized by ethylene oxide (EtO), since it is approved by ISO 14937:2009 standard [57]. Scaffolds were packed in self-seal sterilization pouches (Surgical Grade Paper ThermoSealing + Pet film/PP Coex, CIPAMED, Brazil) and sent to the company ACECIL (Campinas, São Paulo, Brazil) to be sterilized by ethylene oxide. The operational ranges of main parameters were: temperature of 45–50 °C, gas concentration of 450–470 mg/L and exposure time of 24 h. After EtO sterilization, scaffolds were stored at room temperature until in vitro and in vivo assays.

### 2.3. Characterization of PLA and PLA-CaP Scaffolds

The scaffolds’ morphology was assessed by Scanning Electron Microscopy (SEM, JEOL JSM 6460 LV, Peabody, MA, USA) operating at 15 KV, coupled with Energy Dispersive Spectroscopy (EDS-Thermo/System Six model 200, Peabody, MA, USA), which evaluated elemental composition. The samples were placed on aluminum stubs with carbon tape, sputtered-coated with a thin gold layer (Emitech, K550, Abington, MA, USA) to avoid electrical charging. Fourier transform infrared spectroscopy (FTIR, Spectrum 100, Perkin Elmer spectrometer, Shelton, CT, USA) was performed in the range of 4000–550 cm^–1^, 32 scans, and resolution 4 cm^−1^, using the ATR (attenuated total reflectance) mode. The crystalline phase of CaP on coated PLA scaffolds was assayed using X-ray diffraction (XRD). Data were collected with a Brüker D8 Discover diffractometer (Madison, WI, USA), using Cu Kα radiation, 2θ = 3° to 60° with a scan speed 1.23°/min. For XDR analysis, PLA and PLA-CaP dense, square samples were produced. To evaluate the presence of the formation of a CaP coating onto the surface of the PLA-HA scaffolds, a thermogravimetric analysis (TGA) was performed with a Simultaneous Thermal Analyzer (STA) 6000 (Perkin Elmer, Shelton, CT, USA), with a heating rate of 10.00 °C/min, from 22 °C to 700 °C, synthetic air atmosphere, a flow rate of 30 mL/min, on platinum support. The compressive properties of uncoated PLA and PLA-CaP scaffolds were measured in compression in an Instron 33R 5567 tester (Instron, São José dos Pinhais, PR, Brazil), with a 5 kN load cell and crosshead speed of 1 mm/min. For compressive analysis, PLA and PLA-HA, porous scaffolds with 4 mm in axis *z*-axis, 6 × 6 mm in x and y-axes, respectively) were produced. Samples were loaded until 50% strain.

### 2.4. Dimensional Deviation and Porosity

To evaluate if variations occurred in the scaffolds’ dimensions with the CAD model used, scaffolds were measured (n = 5) with a digital caliper in all dimensions. The percentage porosity was estimated as ratio between volume of pores within the scaffolds, V_P_, and the volume of the scaffolds, *V_T_*, by following the ASTM F2450—18 Standard Guide [58] (Equation (1)).

Porosity (%) = (*V_P_*/*V_T_*) × 100 (1)

Scaffolds volume, *V_T_*, was calculated by Equation (2) using the external dimensions of printed parts: diameter (*d*) and height (*h*). While an estimative of the volume of pores, *V_P_*, was obtained using Equation (3).

(2) $$V_{T}=\frac{\mu \times d^{2}}{4}\times h$$

(3) $$V_{P}=V_{T}-\frac{m_{S}}{\rho_{S}}$$

where *m_s_* is the mass of scaffolds and $\rho_{S}$ is density of scaffolds.

The Archimedes principle was employed to determine the density of scaffolds, $\rho_{S}$. A hydrostatic balance was used to measure the dry mass, $m_{dry}$, and the apparent mass of scaffolds after immersion in ethanol, $m_{wet}$. Density ($\rho_{S}$) was calculated by Equation (4), the density of ethanol, $\rho_{ethanol}$, as 0.79 g/cm^3^ at the experiment’s temperature.

(4) $$\rho_{S}=\frac{m_{S}\times \rho_{ethanol}}{\left(m_{dry}-m_{wet}\right)}$$

### 2.5. In Vitro Biological Evaluation

#### 2.5.1. Cytocompatibility Assay

Before assessing cell-surface interactions, a standardized cytocompatibility assay was performed according to ISO10993-5:2009 [59] in order to determine if the scaffolds could indirectly affect cell viability. PLA and PLA-CaP scaffolds were immersed in DMEM (Dulbecco’s Modified Eagle Medium) at 200 mg/mL ratio and incubated for 24 h at 37 °C/5% CO_2_ to produce conditioned media for indirect cell exposure.

Primary human Osteoblasts (HOb) were obtained from the collection of the Clinical Research Unit of Antônio Pedro University Hospital -UFF (UPC-HUAP-UFF, Niterói, RJ, Brazil) (Ethics Committee Approval 57080116.0.0000.5243). Cells maintained in DMEM containing 10% fetal bovine serum (FBS) and 1% penicillin/streptomycin at 37 °C/5% CO_2_ were trypsinized and subcultured in 96-well plates at a density of 1.0 × 10^3^ cells for 24 h until subconfluence. The culture media was replaced with the conditioned media of each scaffold type in quintuplicates, and cells were exposed for 24 h at 37 °C/5% CO_2_. Media conditioned with biocompatible high-density polystyrene beads or fragments of latex, at a 200 mg/mL ratio, were employed as a negative and positive control for cytotoxicity, respectively. Cell viability was assessed with a commercial XTT assay kit (InCytotox, Xenometrix, Allschwil, Switzerland), performed according to the manufacturer, with cell viability related to the Optical Density (OD) measured with a Synergy II Microplate Reader (Biotek Inst., Winooski, VT, USA) at 405 nm.

#### 2.5.2. Production of the 3D Osteoblasts Aggregates (Osteospheres)

The 3D model of human primary osteoblasts was produced through a protocol modified from Restle et al. (2015) [49]. HOb cells were seeded on a density of 5 × 10^3^ cells per well into a 96 well conical bottom plate covered by 1% sterile agar and maintained in 150 uL of medium supplemented with 10% of FBS. Cells were incubated for 4 days at 37 °C/5% CO_2_ on an orbital shaker set at 250 rpm, for the formation of aggregates. These had a mean diameter of 320 ± 38 micrometers and an aspect ratio of 1.05 ± 0.1 (Height/Length), indicating a regular spheroid shape (osteospheres). After seven days of culture, the number of viable cells within the spheroids was measured by trypan blue counting after disaggregation with trypsin/collagenase always remained near 5 × 10^3^ cells (data not shown), indicating the stability of the model during the experimental times of this study.

#### 2.5.3. Exposure of Osteospheres to 3D PLA and PLA-CaP Scaffolds

To simulate the interactions with protein-containing complex biological media, samples of both groups of scaffolds (PLA and PLA-CaP) were pre-treated with inactivated fetal bovine serum by immersion on 300 mL of pure FBS for 24 h. Therefore, four experimental groups were investigated with in vitro assays: PLA, PLA-CaP, PLA+FBS, and PLA-CaP+FBS. Scaffolds from each group were placed in a 24 well plate (one scaffold per well), and a total of eight aggregates were seeded on different points of the surface of each scaffold. The plate was incubated for 7 days in 500 µL of DMEM supplemented with 10% of SFB at 37 °C/5% CO_2_. The culture medium was changed on the 4th day of culture.

#### 2.5.4. Assessment of Cell-Surface Interactions

For the assessment of cell-surface interactions through fluorescence microscopy, the samples were fixed in 4% paraformaldehyde, washed with PBS, and then exposed for 10 min to an ammonium chloride solution. The samples were washed with PBS, and then cells had their membranes permeabilized with treatment with 1% Triton X-100 for 10 min. After washing with PBS, samples were immersed in 3% bovine serum albumin for 10 min and then stained with Phalloidin conjugated with Fluorescein isothiocyanate (FITC) (1:100) for cytoskeleton labeling for 30 min and 1:5000 DAPI (4′,6-diamidino-2-phenylindole), a nuclear marker, for 30 min. The samples were stored in a DABCO (1,4-diazabicyclo [2.2.2]octane) solution.

The cell density on the different scaffolds was compared in the different scaffolds from two parameters: total protein content and the release of the cytoplasmic enzyme lactate dehydrogenase (LDH) after total cell permeabilization. At 7 days, scaffolds were washed with PBS 1x, then exposed to 200 µm of serum-free DMEM containing 1% Triton X-100, and incubated for 24 h. The samples were diluted in PBS for decreasing the Triton X-100 concentration from 1% to 0.01%.

A sample of 5 µL of each culture media was used to evaluate the total released protein with a Bradford assay kit (Protein Assay, Biorad, Hercules, CA, USA). Optical density was measured at 595 nm using a Sinergy II Microplate Reader (Biotek Instruments, Winooski, VT, USA. The total amount of protein after permeabilization was subtracted from the protein quantified before treatment with triton X100. A sample of 20 µL of each well was transferred to another plate and added with 240 μL LDH II and LDH III solutions (In Cytotox, Xenometrix, Allschwil, Switzerland). Optical density was measured at 540 nm using a Sinergy II microplate reader (Biotek Instruments, Winooski, VT, USA) at kinetic mode for 25 min at 37 °C.

#### 2.5.5. Release of Biological Mediators

The release of some different biological mediators of interest to tissue engineering by the exposed osteospheres was assessed of each scaffold/treatment culture media. The quantification of the release of the basic Fibroblasts Growth Factor (FDF2), Platelet-Derived Growth Factor (PDGFbb) and the Vascular Endothelial Growth Factor (VEGF) was performed using an Enzyme-Linked Immunosorbent Assay (ELISA) with standard ABTS ELISA Development Kits (PeproTech, Rocky Hill, NJ, USA). The procedure followed the recommendations of the manufacturer. The reactions were stopped with a 5% sodium dodecyl sulfate (SDS), and the OD was measured in a Synergy II microplate reader (Synergy II, Biotek Instruments, Winooski, VT, USA) at 405 nm with wavelength correction set at 650 nm. The activity of Alkaline Phosphatase (ALP), a marker of the early stages of osteoblast maturation, was measured with a Kinetic Alkaline Phosphatase kit (Bioclin, Rio de Janeiro, RJ, Brazil), in which the enzyme causes para nitrophenyl phosphate (PNPP) hydrolysis, and this process is measured colorimetrically. After incubation, the scaffolds were washed with PBS 1x, then exposed to 200 µL of a medium solution without SFB with 1% Triton X-100 and incubated for 24 h. 60 μL of the samples were transferred to a 96 well microplate, and 200 µL of reagent was mixed per well. The plate was incubated for 3 h, and two readings were taken every 30 min at 37 °C at 405 nm.

### 2.6. In Vivo Study

#### 2.6.1. Ethical Aspects

The animal breeding and procedures followed the conventional guidelines of the NIH Guide for the Care and Use of Laboratory Animals, the Brazilian Directive for the Care and Use of Animals for Scientific and Didactic Purposes—DBCA, and the Euthanasia Practice Guidelines of the CONCEA (Brazilian National Council for the Control of Animal Experimentation). The Ethics Committee of Animal Use from Fluminense Federal University (CEUA/UFF, Niterói, RJ, Brazil) approved this work’s research protocol (protocol number #934). The present study’s report considered the ARRIVE guidelines concerning the relevant items [60] supplemented by PREPARE [61].

#### 2.6.2. Welfare of Animals

Before and after surgical procedures, the animals were kept in individual cages and under standard conditions (water ad libitum and regular rat pellets). The ambient temperature was maintained between 16 and 20 °C, being ideal for the growth of the animals and photoperiod control of 12 h light and 12 dark hours was established for the development of the complete metabolic cycle.

#### 2.6.3. Experimental Groups

Forty-five Wistar rats, weighing between 300 and 400 g, were randomly divided into three experimental groups as follows: PLA, PLA-CaP, and PLA-CaP plus 5 μh rhBMP2 (Infuse^®^ Bone Graft Small Kit, Medtronic Spinal and Biologics, Memphis, TN, USA) (PLA-CaP-BMP2) (n = 15). The scaffolds implantation was conducted following ISO standard 10993-6 [62]. The animals were also randomly divided into three experimental periods of 1, 3, and 6 months (n = 5).

#### 2.6.4. Anesthesia and Surgical Procedures

The animals were deprived of the solid diet six hours before the surgery and submitted to general anesthesia, receiving as anesthetic medication a solution composed of 100 mg/kg of Ketamine (Francotar^®^—Virbac), 10 mg/kg of Xylazine (Sedazine^®^—Fort Dodge) and 5 mg/Kg of Midazolam (Roche) intramuscularly.

**Figure 2:**
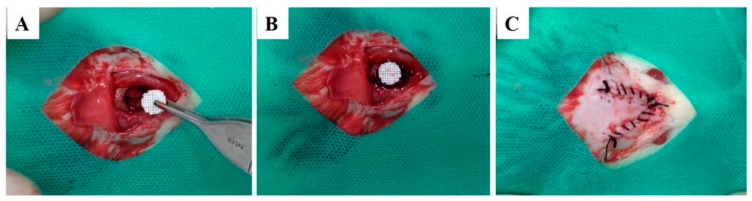
Surgical procedures for scaffold implantation in 8 mm calvaria defect. Surgical bone defect (**A**), scaffold implantation in situ (**B**) and defect sutured (**C**).

After general anesthesia and observing the absence of reflexes to the pain, the animals were positioned in a ventral decubitus, trichotomized in calvaria, and submitted to the operative field’s antisepsis with 2% non-alcoholic chlorhexidine. Subsequently, a semilunar incision was made on the calvaria using blade #15 C (Becton-Dickinson^®^, Curitiba, PR, Brazil). Then, with the aid of a periosteum detachment (Molt, Duflex, Barueri, SP, Brazil), the periosteum was removed. The bone defect was performed with a trephine bur with 8 mm of internal diameter, mounted in contra-angle with reduction of 16:1, was coupled in surgical motor, at 1500 rpm, under constant irrigation with physiological solution. Finally, the scaffold was implanted on bone defect (Figure 2), the flap repositioned and sutured with interrupted stitches with 5.0 nylon thread (Ethicon^®^, Johnson & Johnson). Subsequently, the surgical field antisepsis was performed with gauze and 2% chlorhexidine non-alcoholic solution. It was administrated 1 mg/kg, subcutaneously every 24 h for three days, starting on the day of surgery and maintained for two more days. The animals were euthanized 1, 3, and 6 months’ post-surgical procedures by applying a lethal dose of general anesthetic (Thiopental 150 mg/kg).

### 2.7. rhBMP-2 Quantification on PLA-CaP Scaffolds

The levels of rhBMP-2 were quantified by use of a commercially available enzyme-linked immunosorbent assay (ELISA) kit rhBMP-2 Human BMP2 DuoSet ELISA (R&D Systems) according to manufacturer’s protocol. A solution of 40 mM Tris-Cl containing 500 nM NaCl pH 7.2 buffer was used to elute the BMP2 overnight at 4 °C from the PLA-CaP scaffolds from in vivo study. A 96-well plate was pre-coated with BMP2 capture antibody and incubated overnight at room temperature. After this step, control standard, and samples were added per well and the plate was incubated for two hours at room temperature. This step was followed by aspirated, washing with 0.05% Tween-20 in phosphate saline buffer pH 7.2–7.4 wash buffer (PBS-T), and removing it. rhBMP-2 capture antibody was added to each well and plates were incubated for two hours at room temperature. In sequence, wells were rewashed as described above with PBS-T, and substrate solution (Pierce TMB Substrate Kit) was added to each well. The plate was incubated for 20 min at room temperature (protected from light) and the reaction was interrupted with Stop Solution (2N H_2_SO_4_). Each well’s optical density was determined immediately using a microplate reader (SpectraMax Paradigm, Molecular Devices, San Jose, CA, USA) set to 450 nm. rhBMP-2 concentrations of samples were determined from the optical densities in relation to standard experimental curves (four-parameter logistic, 4-PL, curve-fit). The 40 mM Tris-Cl containing 500 nM NaCl pH 7.2 buffer was used as blank and no interference was detected. All samples were measured twice. The mean level of each measurement was used for analysis.

### 2.8. Histological Processing

The bone blocks containing the scaffolds were collected according to the protocol of the Applied Biotechnology Laboratory/Federal Fluminense University (LABA/UFF, Niterói, RJ, Brazil). The decalcification of calvarias was achieved with 10% buffered ethylenediaminetetraacetic acid (EDTA) for 2 days at room temperature before the histological processing. The samples were fixed in 4% buffered formalin (pH 7.4) for 48 h and washed in running water for one hour, dehydrated in crescent alcohol solutions, diaphanized in xylol, and included in paraffin. At the end of the processing, they were cut in a microtome (Jung-Leica RM 2054, Nussloch. Germany) with 5 μm thickness and stained with hematoxylin-eosin (HE) for descriptive evaluation of newly formed bone, the pattern of scaffolds, and connective tissue.

For the descriptive histological analysis, a Light Field Light microscope (OLYMPUS^®^ BX43, Tokyo, Japan) was used. Captures of the selected images were performed through a microscope-coupled camera (OLYMPUS^®^ SC100, Tokyo, Japan), associated with high-resolution software (CELLSENS^®^1.9 DigitalImage, Tokyo, Japan).

### 2.9. Histomorphometric Analysis

Each histological calvaria slide was examined under a light microscope (OLYMPUS BX43, Tokyo, Japan). Five non-superimposing photomicrographs were captured at 40× magnification using a high-resolution digital camera (OLYMPUS BX43, Tokyo, Japan), to the regions surrounding and into the implanted biomaterials. The histomorphometric evaluation was performed using Image-Pro Plus^®^ 6.0 software (Media Cybernetics, Silver Spring, MD, USA) which generates a grid of 250 points that allowed for evaluating the volume density of newly formed bone, biomaterial (scaffold), and connective tissue. The values were stored in a database developed using Microsoft Excel^®^ spreadsheet software.

### 2.10. Statistical Analysis

For the in vitro assessment, the results from three biological assays for each scaffold type and treatment (PLA, PLA-CaP, PLA+FBS, and PLA-CaP+FBS) were compared through the analysis of variance. A D’Agostino Pearsons normality test was performed, followed by Kruskal-Walis non-parametric tests with Dunn ad hoc post-test. Significance was considered at 5% of alpha error. All statistical analyses were performed using GraphPad Prism 7.0 Software (GraphPad Inc., San Diego, CA, USA).

In the in vivo assessment, a quantitative description of the volume density of newly formed bone, biomaterial (scaffold), and connective tissue was done by parametric description with a *p*-value of < 0.05 considered significant. The obtained data were not normal (Shapiro-Wilk normality test); they were transformed in Y logarithm, and a *p*-value < 0.05 was considered significant. ANOVA and Tukey’s post-tests were used to investigate the differences between groups at the same experimental period and differences between the same group at different times. The calculations were performed using Prism Graph Pad 7.0 software (Inc., La Jolla, CA, USA).

## 3. Results

### 3.1. Characterization of PLA and PLA-CaP 3D Scaffolds

The uncoated PLA and PLA-CaP scaffolds were evaluated by optical microscopy, as shown in Figure 1. The printed scaffold presented a morphology compatible with the projected CAD model (Figure 1 A,B). It was possible to design a scaffold suitable for bone tissue engineering, with interconnected pores. Also was seeing that the alkaline hydrolysis effectively removed the possible non-projected filaments. The porosity of uncoated PLA and PLA-CaP scaffolds was found to be nearly 50%, with no difference between the groups. The pore volume for each was in the same range and indicated that CaP coating does not interfere with the scaffold architecture (Table 1). Under compressive force, uncoated PLA and PLA-CaP scaffolds showed a plastic response with ~20 MPa of compressive strength, similar to trabecular bone (Table 2). Similarly, the elastic modulus, determined from the slope of the stress vs. strain curve, was ~0.5 GPa, also similar to trabecular bone.

**Figure 3:**
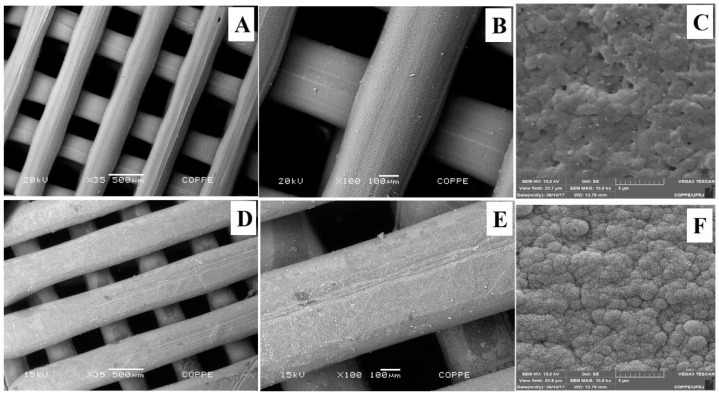
SEM micrographs of scaffolds before (**A**–**C**) and after CaP coating (**E**–**G**).

The SEM micrographs of the 3D scaffolds are shown in Figure 3. CaP biomimetic coating was effective, forming a continuous and uniform bone-like apatite layer over the entire surface of PLA (Figure 3E,F). This behavior indicates that the number of nucleating sites on the polymer’s surface during the first hours of immersion was high, favoring the formation of a dense and uniform CaP layer. The homogeneity of CaP layer is directly related to the presence of nucleating sites during the first hours of SBF immersion. These bioactive sites may result from the alkaline hydrolysis, which incorporates OH-groups, tending to facilitate the Ca^2+^ deposition onto the surface [64].

**Figure 4:**
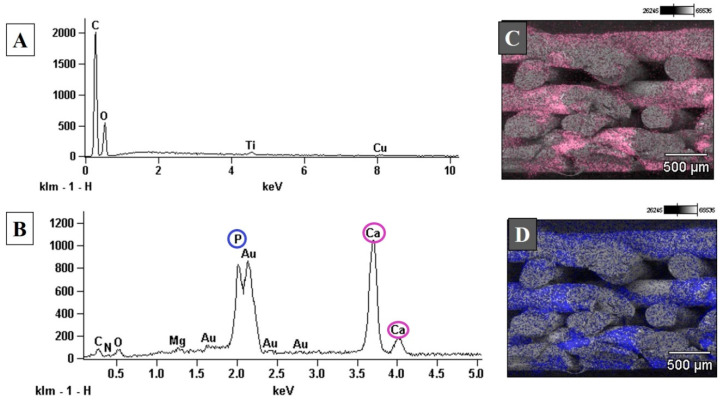
EDS spectrum of the top surface uncoated PLA (**A**) and PLA-CaP (**B**) scaffolds. Element mapping image for calcium, imaged in pink, (**C**) and phosphorous, imaged in blue, (**D**) of the fracture surface of PLA-CaP scaffold.

The elemental composition of uncoated PLA scaffolds, accessed by the EDS spectrum, showed that the PLA’s main elements were carbon and oxygen, as expected (Figure 4). After biomimetic coating, it is possible to see the presence of calcium and phosphorous peaks, suggesting an effective deposition of apatite on the scaffold’s surface. A small titanium peak was also observed in uncoated PLA scaffolds (Figure 4A). It can be related to titanium dioxide (TiO_2_), present in the composition of a wide range of white pigments commonly used in various industries [65]. Element maps illustrating the distribution of calcium (Figure 4C) and phosphorous (Figure 4C) on the fracture surface of PLA-CaP scaffolds showed a uniform coating throughout the thickness of the scaffolds.

**Figure 5:**
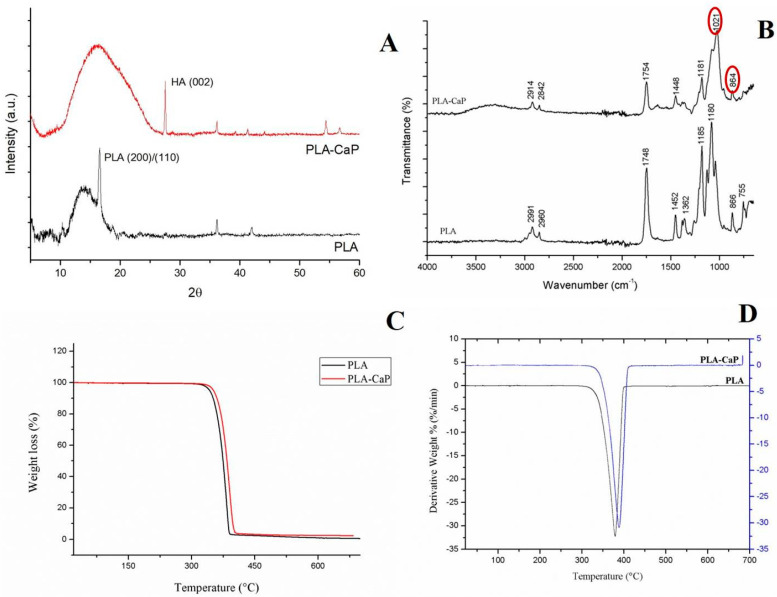
XRD (**A**) and FTIR (**B**) spectra of PLA and PLA-CaP scaffolds. TGA (**C**) and DTG (**D**) curves of uncoated PLA and PLA-CaP scaffolds exhibited single-step decomposition.

A structural characterization was carried out by XRD to assess the deposition of bone-like apatite by SBF. XRD diffractions of uncoated PLA and PLA-CaP scaffolds are shown in Figure 5A. The XRD pattern showed peaks in 2θ = 16° and 27°, indicated characteristic peaks of PLA [66,67]. After biomimetic coating, peaks in approximately 26°, 36°, 40°, and 54° correspond to the (002) plane, (301) plane, (221) plane, and (104) plane of apatite, respectively [68]. The Ca/P molar ratio found in PLA-CaP scaffolds was 1.49 ± 0.34, corresponding to nonstoichiometric apatite; in other words, a calcium-deficient hydroxyapatite (CDHA) [69,70].

The FTIR spectra of the uncoated PLA and PLA-CaP scaffolds are presented in Figure 5B. Peaks located at 2921 cm^−1^ and 2842 cm^−1^ corresponded to asymmetric and symmetrical vibrations of the CH_2_ group, respectively. A characteristic band of PLA was found at 1746 cm^−1^, corresponding to the vibration of the carbonyl group [71,72]. PLA-CaP spectrum shows the presence of phosphate (1021 cm^−1^) and carbonate groups (867 cm^−1^), confirming the deposition of apatite coating [73,74]. A peak at 1448 cm^−1^ in PLA-CaP suggests that the apatite obtained is carbonate-containing hydroxyapatite (CO_3_^2−^ ion).

The thermal properties of uncoated PLA and PLA-CaP scaffolds were investigated by TGA (Figure 5C,D). The derivative curves of mass loss (DTG) are shown in Figure 5D. Considering the amount of the residues obtained in TGA analysis, it was possible to infer that approximately 2% wt of apatite was deposited onto the scaffolds. Both DTG curves exhibit a single-step of decomposition, where the main degradation temperature increased with the deposition of apatite (from 379.47 °C in uncoated PLA to 384.86 °C in PLA-CaP).

### 3.2. In Vitro Evaluation

**Figure 6:**
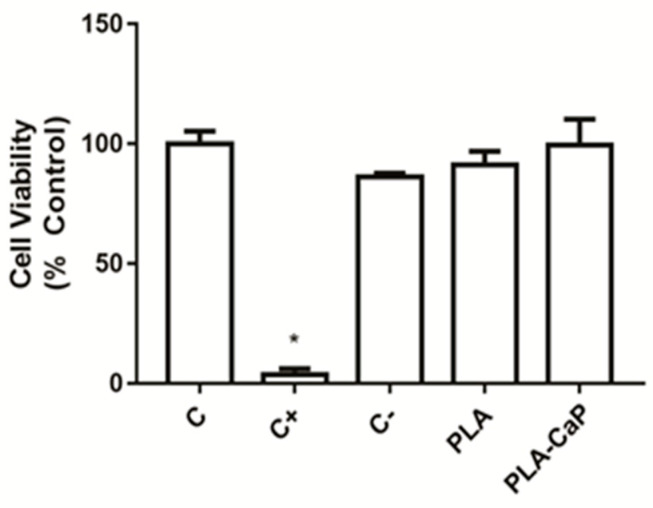
Cytocompatibility evaluation of the scaffolds, as evaluated by XTT assay. Bars indicate mean ± SD results normalized as a percentage of the unexposed control group (C). Extracts of polystyrene beads were used as negative control (C−) and fragments of latex as a positive control (C+). An asterisk indicates a significant difference with all other groups (*p* < 0.05).

Figure 6 shows the result of a standardized cytotoxicity assay of uncoated PLA and PLA-CaP scaffolds extract with human primary osteoblasts. It is possible to observe that only the positive control (latex) was able to induce high levels of cytotoxicity, while both scaffolds presented responses similar to the negative control, without significant difference (*p* > 0.05) from the experimental control (100% of viability).

**Figure 7:**
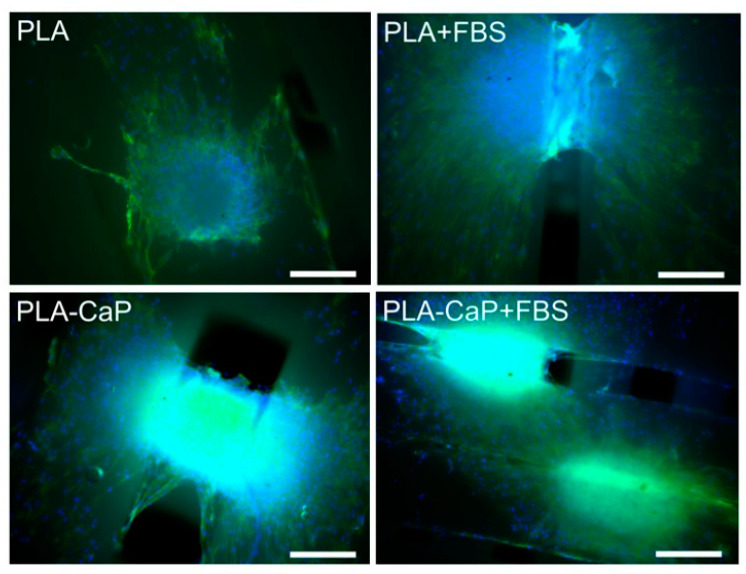
Fluorescence microscopy images of HOb osteospheres cultivated for 7 days over surfaces of PLA, PLA-CaP scaffolds, pre-treated or not with FBS. Cell nuclei appear in blue, and actin microfilaments marked in green. Images obtained either with ×10 objectives. Bars indicate 200 µm.

HOb spheroids were then cultured over the scaffolds for seven days, and cell-surface interactions were assessed by fluorescence microscopy, as shown in Figure 7. Osteospheres seeded over uncoated PLA scaffolds remained well defined, presenting a similar size from the original aggregates (around 300 mm of diameter), even though several cells either migrated or proliferated from the aggregate into the material’s surface on the vicinity of the spheroid. A similar interaction was observed for uncoated PLA and PLA-CaP scaffolds pre-treated with FBS, but with increasing evidence of aggregate disassembly and colonization of the material surface. Such a pattern seems to reach its maximum in PLA-CaP surfaces pre-treated with FBS, where the remaining aggregates appear less dense and more spread, with the scaffold filaments completely covered with cells at greater distances from the original osteosphere. Figure 6 also shows that cells from some spheroids interacted with rifts from the scaffold’s lattice, starting to colonize inner portions of the material’s structure. In all scaffolds, osteoblastic cells predominantly presented a fusiform morphology compatible with a proliferative phenotype.

**Figure 8:**
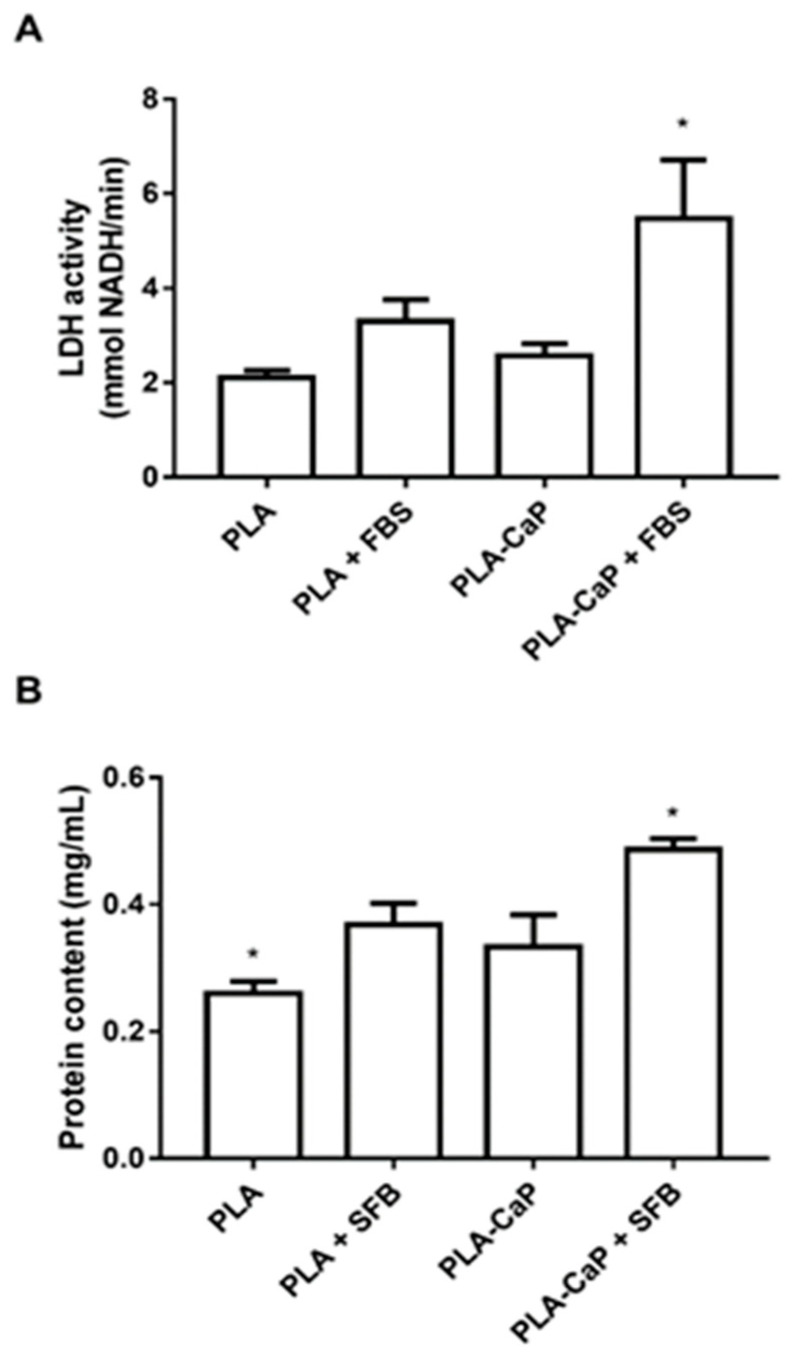
Estimative of the adhered cells to each scaffold, as measured by the release of cytoplasmic lactate dehydrogenase (**A**) or total protein content (**B**), after solubilization with the detergent Triton X-100. Bars indicate the mean and SD of NADH reduction in culture media 25 min after the addition of substrates and solubilizer. The asterisk indicates significant difference from all groups (*p* < 0.05, Kruskal-Wallis Test with Dunn post-test).

Total cell and spheroid over each surface were estimated through the detection of LDH, and total protein content after cell permeabilization (Figure 8). While there was no significant difference between uncoated PLA and PLA-CaP scaffolds, it was possible to observe, from both parameters, that PLA-CaP+FBS had a significant (*p* < 0.05) presence of cells observed in pure PLA.

**Figure 9:**
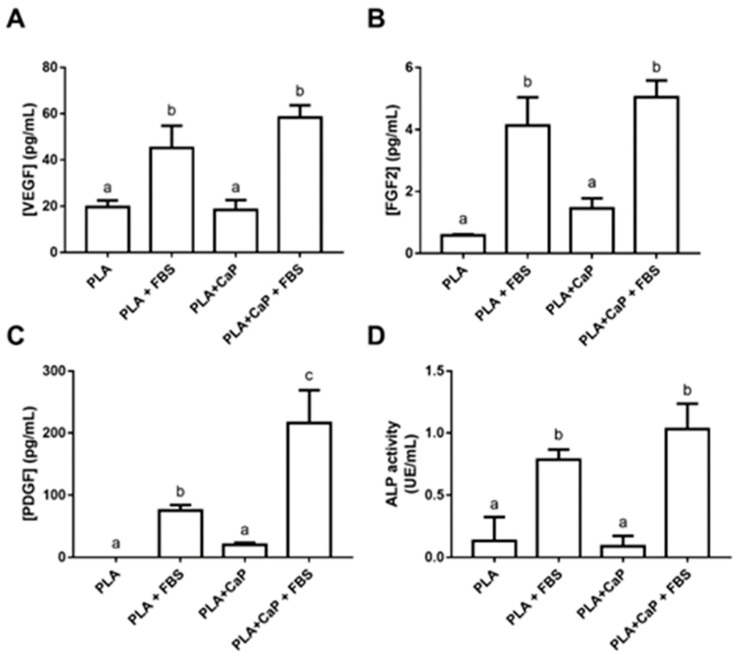
Evaluation of the release of biological markers into the culture media by human osteoblast spheroids after 7 days incubation inside the tested scaffolds. (**A**). Concentration of released Basic Fibroblastic Growth factor (FGF-2). (**B**). Concentration of released Vascular Endothelial Growth Factor (VEGF). (**C**) Concentration of released Platelet-Derived Growth Factor (PDGF). (**D**) Alkaline Phosphatase (ALP) activity. Bars indicate mean and standard deviation of three biological replicates with five technical replicated each. Bars with different letters are significantly different (*p* < 0.05). UE: units of enzyme activity.

Figure 9 shows the biological mediators secreted by the HOb cells and spheres loaded in the scaffolds after seven days. The analysis of released FGF-2, PDGF, and VEGF shows that groups pre-treated with serum presented higher levels of secreted growth factors (*p* < 0.05). In the case of PDGF, the FBS-treated biomimetic coating induced close to a threefold increase when compared to uncoated PLA + FBS (Figure 9C). Alkaline phosphatase (ALP), an important biomarker of the initial steps of osteoblast engagement on mineralization, was measured in cells adhered to the scaffolds after permeabilization with triton X100 (Figure 9D). Similar to the growth factors, the ALP secretion was significantly increased in the FBS-treated groups (even after the removal of background activity), with the higher activities observed with PLA-CaP-FBS (1.03 UE/mL).

### 3.3. Quantification of rhBMP2 in the PLA-CaP+BMP2 Scaffolds

The incorporation of rhBMP-2 into the PLA-CaP scaffolds was determined by ELISA, obtaining the levels of 13.5 µg/mL, very similar to those found on FDA-approved absorbable collagen sponges (ASC) for orthopedic trauma and oral maxillofacial applications [51].

### 3.4. Descriptive Histological Evaluation

**Figure 10:**
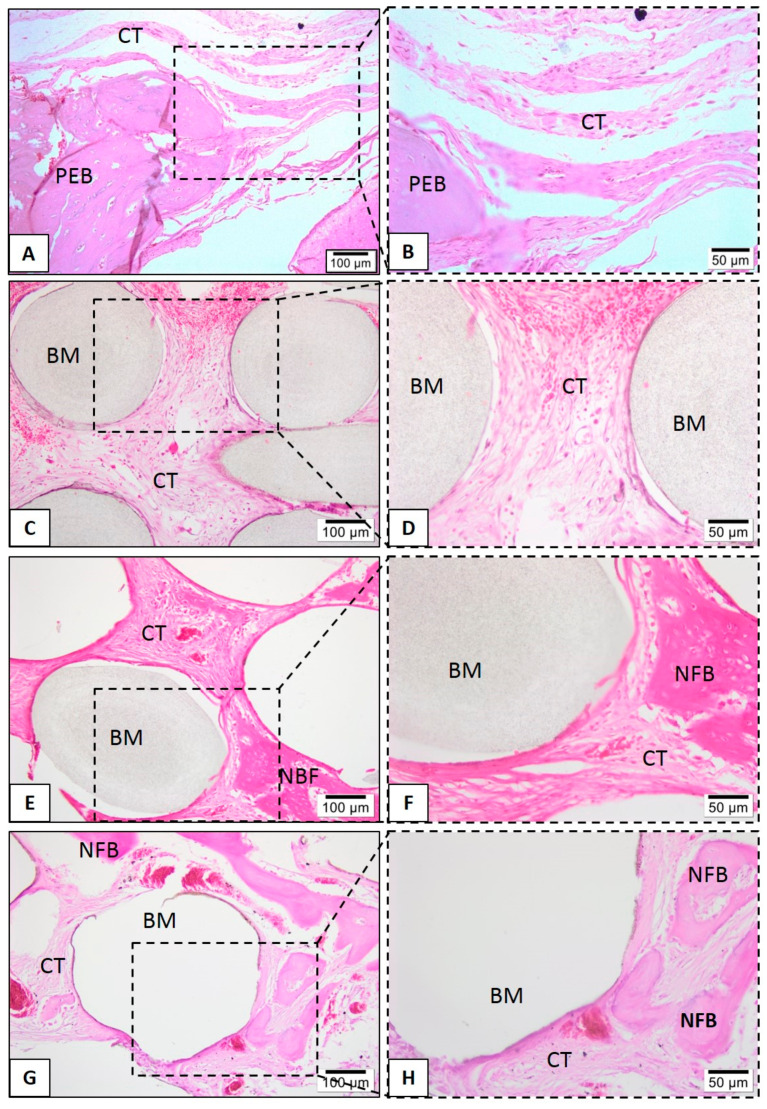
Representative photomicrographs of calvaria defect after 1 month. Histological section stained with hematoxylin/eosin from the region of control without biomaterial (**A**,**B**), biomaterial implantation in the PLA (**C**,**D**), PLA-CaP (**E**,**F**) and PLA-CaP-rhBMP-2 (**G**,**H**) groups—1 month after implantation. The region occupied by pre-existing bone is indicated with (PEB), newly formed bone (NFB); connective tissue (CT) and biomaterial/PLA scaffold (BM). Results are representative of 5 rat/group.

Histological evaluations were performed to assess the biological response to the tested biomaterials. None of the tested scaffolds lost their structural integrity, and no significant degradation after the in vivo assay was found. Defects that were filled with neat PLA, at 1 month, showed connective tissue interspersed with biomaterial and rare inflammatory cells (Figure 10D). However, after six months, the neat PLA group presents small bone formation levels, present from the peripheries to the center of scaffolds (Figure 10C,D).

**Figure 11:**
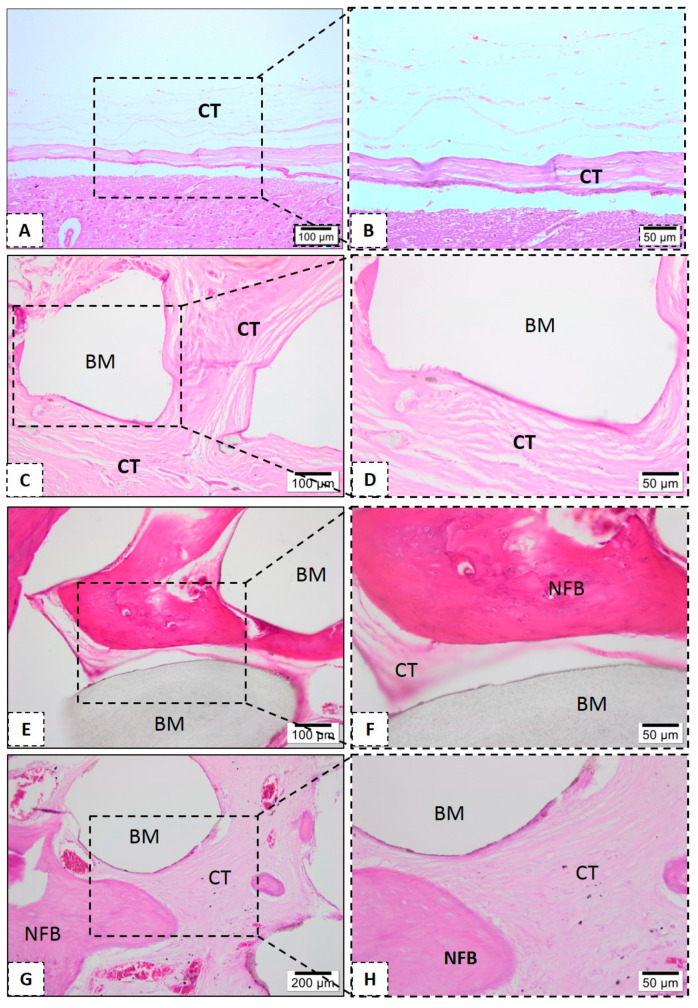
Representative photomicrographs of calvaria defect after 3 month. Histological section stained with hematoxylin/eosin from the region of control without biomaterial (**A**,**B**), biomaterial implantation in the PLA (**C**,**D**), PLA-CaP (**E**,**F**) and PLA-CaP-rhBMP-2 (**G**,**H**) groups—3 month after implantation. The region occupied by pre-existing bone is indicated with (PEB), newly formed bone (NFB); connective tissue (CT) and biomaterial/PLA scaffold (BM). Results are representative of 5 rat/group.

**Figure 12:**
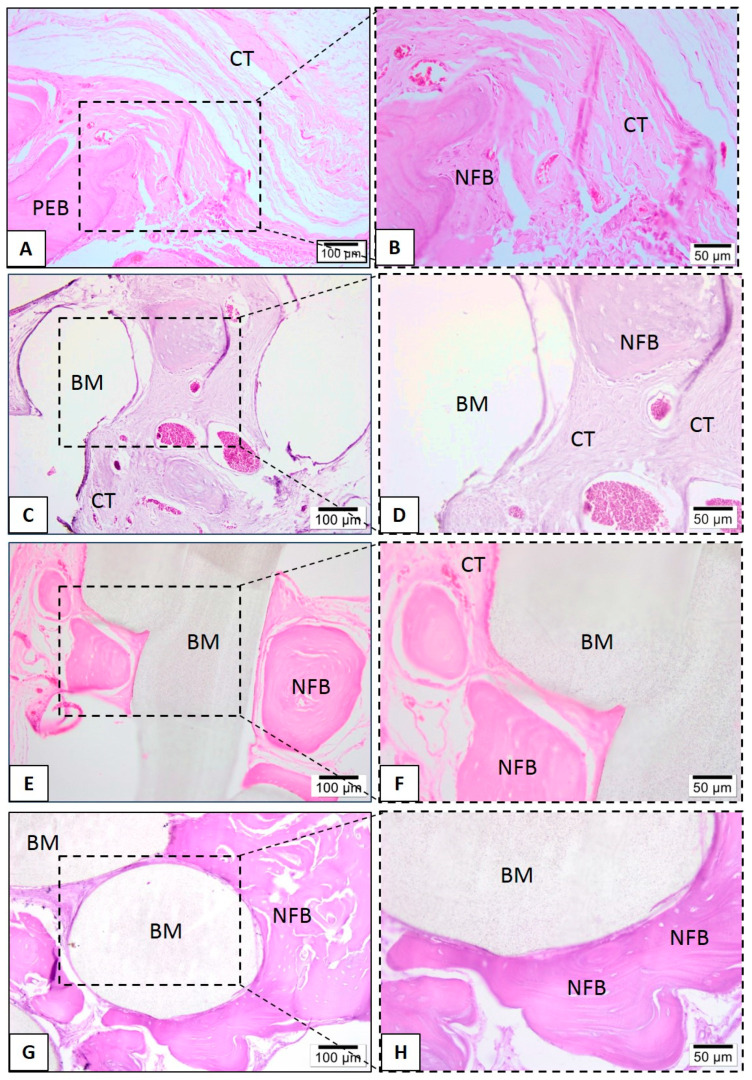
Representative photomicrographs of calvaria defect after 6 months. Histological section stained with hematoxylin/eosin from the region of control without biomaterial (**A**,**B**), biomaterial implantation in the PLA (**C**,**D**), PLA-CaP (**E**,**F**) and PLA-CaP-rhBMP-2 (**G**,**H**) groups—6 months after implantation. The region occupied by pre-existing bone is indicated with (PEB), newly formed bone (NFB); connective tissue (CT) and biomaterial/PLA scaffold (BM). Results are representative of 5 rat/group.

In the PLA-CaP and PLA-CaP-rhBMP-2 groups, in the first month it was possible to observe immature bone trabeculae with some osteocytes distributed throughout the bone defect (Figure 10). At month 3, both present large mature bone trabeculae, with more organized bone tissue (Figure 11E–H). At month 6, the PLA–CaP-BMP2 group presents bone tissue formed with a more advanced degree of maturity and large area, as compared to the PLA-CaP group (Figure 12). Bone tissue surrounded the PLA-CaP-BMP2 scaffolds, with an increased aspect of bone repair. After 6 months, both scaffolds remained inside the defect, with structural integrity and no significant degradation.

### 3.5. Histomorphometric Evaluation

**Figure 13:**
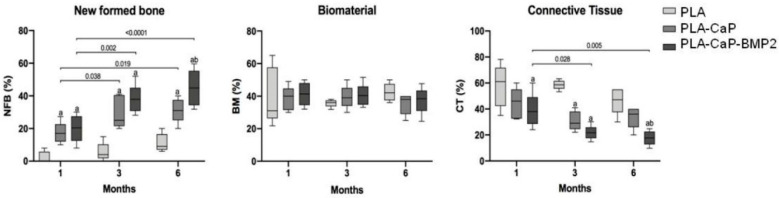
Box plot comparing the volume of new formed bone (NFB), biomaterial (BM) and connective tissue (CT) (%) of PLA, PLA-CaP and PLA-CaP-BMP2 groups (n = 5) after experimental periods. Horizontal bars represent statistical differences between same group at different experimental periods and its respective *p* values. (a) represents significant statistical differences compared to PLA group. (b) represents significant statistical differences compared to PLA-CaP group (ANOVA and Tukey post-test, *p* < 0.05).

The statistical results allowed comparing the progressive newly formed bone, reminiscent biomaterial (scaffolds), and connective tissue in the periods of 1, 3, or 6 months. Regarding the connective tissue, no statistical differences were observed between groups and experimental periods (Figure 13). The newly formed bone parameter in the defects implanted with PLA was around 2.3% (SD ± 3.54) after one month. At the same period, PLA-CaP (17.25%, SD ± 6.4) and PLA-CaP-BMP2 (20.09%, SD± 8.33) showed a significantly higher value than PLA (*p* = 0.01 and *p* = 0.002 respectively). After three months of surgery, this difference was repeated. The PLA-CaP (29.80%, SD ± 9.93) and PLA-CaP-BMP2 (37.88%, SD ± 8.87) presented better values of newly formed bone than PLA (5.66, SD ± 5.61) (*p* < 0.0001). Also, after 3 months, a time-dependent increase of new formed bone at PLA-CaP and PLA-CaP-BMP2 was observed. Six months after implantation, the PLA-CaP (31.2, SD ± 7.39) and PLA-CaP-BMP2 (44.85 SD ± 11.09) scaffolds presented significantly better biological response of newly formed bone compared to the PLA group (11.2, SD ± 5.54). Moreover, in this experimental period, we observed that PLA-CaP-BMP2 (44.85 SD ± 11.09) showed higher values of NFB than PLA-CaP (31.2, SD ± 7.39) (*p* = 0.02). The time-dependent increase was repeated in the groups PLA-CaP and PLA-CaP-BMP2 in comparison with the results from three months.

## 4. Discussion

One of the challenges of current regenerative medicine is the reconstruction of large-sized bone defects caused by diseases, trauma, or tumor resection. The autogenous bone is still considered the international gold standard for bone grafts, but it has significant limitations regarding desired graft size and shape, as well as the need for a second surgical intervention. Aiming to provide alternatives to these autologous materials, different studies have been conducted using polymers and apatite composites to produce scaffolds by 3D printing (or additive manufacturing) technologies [75,76,77,78]. These techniques allow the accurate production of complex scaffolds with well-controlled architecture and high reproducibility, aiming to produce personalized grafts based on micro-computed tomography scans of the patient’s defect. In this context, the PLA scaffolds produced in this work presented pore size of 500 μm, 50% porosity, and mechanical properties similar to trabecular bone. These parameters are requirements for the successful permeability of nutrients and tissue growth [79]. However, since PLA presents low bioactivity, surface modification is needed to guarantee suitable cells interaction to it. Using FDM technology, the present work investigates a viable alternative to create biomaterials with adequate morphological, physical, and biological properties for bone substitution by using a biodegradable polymer coated with a biomimetic apatite.

For resorbable bone substitutes, it is expected that the implanted biomaterial must be able to stimulate cell interaction and colonization of the implant surface, metabolizing the biomaterial at the same time that they produce a new matrix, allowing osseointegration. Andu et al. [80] reported several advantages of 3D printed PLA scaffolds in comparison to the particulate graft tested in a continuous defect in an animal model. Nevertheless, PLA was reported as with low bioactivity and, therefore, the treatment of its surface with apatite is an interesting and well-known alternative [81]. Biomimetic coating treatment was performed with the aim to induce a thin and homogeneous layer of hydroxycarbonated apatite, promoting a bioactive material. The use of SBF solutions presents a simple technique, which results in a bone-like apatite mineral coat on surface of polymer [82]. Indeed, the use of SBF solution for 14 days promoted the deposition of a homogeneous layer of apatite on the polymeric scaffold. Higher magnification micrographies show that the morphology of the formed apatite was a flake-like assembly, while EDS analysis indicated that calcium and phosphorous were the main elements. Moreover, the biomimetic methodology used was efficient to uniformly coat all the scaffolds surface, even the internal struts. A recent study (Jaidev and Shatterjee, 2019) [83] showed that a biomimetic coating of 3D printed PLA scaffolds can be produced through a surface functionalization with polyethyleneimine (PEI) and citric acid, followed by immersion in SBF, achieving interesting biological responses. Similarly, our results show that a biomimetic coating can also be achieved by the immersion of non-functionalized, NaOH-treated PLA scaffolds. It is important to notice that the alkaline treatment is a very important step in the production of polymeric scaffolds because it is known that it increases their hydrophilicity, roughness, and carboxylic acid density on the surface of polymer matrices, which has a significant impact on cellular adhesion. This process allows the incorporation of carboxylic groups into the polymer chains by breaking the ester bond, increasing the number of active sites for cellular binding to materials, favoring the deposition of apatite by the biomimetic coating, as well removing possible non-projected filaments present in the scaffold.

Similar to in vivo mineralization, the deposition of biomimetic apatite layer on PLA surface has two distinct stages. After surface hydrolysis, mineral nucleation occurs on the polymer surface (primary nucleation). The carboxylic groups generated by alkaline hydrolysis serve as mineral nucleation sites. They can transfer to carboxylate anions at the buffered solution (pH 7.4) and provide a negatively charged surface to adsorb positive calcium ions in the surrounds. The locally excess calcium ions induce apatite nucleation by combining phosphate ions in the vicinity. The next stage is characterized by the additional mineral crystals grow on the nucleated mineral (crystal growth). Apatite continues to grow by consuming calcium and phosphate ions of the SBF solution [55,56].

According to the number of residues identified as the inorganic portion in the TGA analysis, nearly 2%wt of apatite was deposited onto the PLA scaffolds, suggesting a thin layer of biomimetic coating. FTIR spectra have shown significant apatite peaks, and XDR analysis detected that the crystalline phase deposited onto PLA scaffold was composed of hydroxyapatite. EDS graphic indicated a peak of titanium, which may be related to titanium dioxide (TiO_2_), commonly present in industrial white pigments. Nevertheless, the eventual presence of pigment residues did not affect the material’s biocompatibility, as shown by the performed cytotoxicity assay. The elemental composition of PLA-CaP scaffold showed that the Ca/P ratio was 1.49, corresponding to calcium-deficient hydroxyapatite (CDHA), which is easy to obtain with solutions with relatively high concentrations of Ca^2+^ and PO_4_^3−^ such as SBF. The chemical formula of CDHA presents great variability, resulting in an unstable Ca/P ratio.

For the initial assessment of such biological responses, the present study employed an in vitro tridimensional model based on a spheroidal 3D culture of osteoblastic cells, as proposed previously [49]. The use of tridimensional cell aggregates can increase the similarity with the microenvironment present on in vivo tissues, with more information than 2D cells models [84,85], as cells can remain integrated into the spheroid or migrate and proliferate to colonize a material surface, depending on its osteoconductive properties. The primary human osteoblast spheroids also allow the focal seeding of cells in a specific point of contact, providing insights on the migration and extent of occupation of a material surface over time. Recently, Baptista et al. (2018) [48] employed spheroids of adipose-derived stromal/stem cells to observe interactions with 3D-printed composites of PLA and carbonated HA (CHA). The authors discuss the intimated interaction between spheroids and PLA/CHA printed scaffolds, with cell spreading in the material surface. In the present study, the 3D model enabled observing that NaOH-treated PLA scaffolds were already able to interact with bone cells, showing some level of adhesion and migration over its surface.

Curiously, no striking differences in these parameters were observed from samples with biomimetic coating, even though a slight but significant (*p* < 0.05) increase in total cell content (as measured by protein quantification) was observed in PLA-CaP. However, interesting results were observed in the presence of a pre-treatment of the surfaces with fetal bovine serum. The serum is a complex mixture of proteins and biomolecules, several of which participate in cell adhesion, migration, and growth. The cell adhesion onto the surface of materials occurs via integrin interaction with bioactive sites on the surface. To partially emulate the in vivo complex interactions of serum proteins and scaffold surfaces, we included a pre-treatment with bovine fetal serum. Interestingly, PLA-CaP added with FBS components showed a more than twofold increase in cell content after 7 days of incubation compared to untreated PLA, and was statistically higher than PLA + FBS, also presenting evidence of increased interactions with human osteoblast spheroids, as shown by fluorescence microscopy. The incorporation of serum proteins such as vitronectin and fibronectin may be a way to increase the number of bioactive sites that are recognized by cells on the surface of materials such as PLA [86].

Alkaline phosphatase activity, an important biomarker of early osteoblasts response, similarly increased in the FBS-treated groups (even after removal of background activity), with the higher levels observed with PLA-CaP+FBS. The increase in ALP activity may indicate a greater response to cell differentiation capacity for PLA-CaP scaffold with FBS and is in accordance with recent results of studies with HA-coated PLA scaffolds in contact with mesenchymal stem cells [84]. The present findings provide insight into the possible role of the biomimetic coating in PLA and the interface with such proteins, resulting in increased interactions with cells. Further studies are required, though, to assess in-depth the participation of specific proteins in this process.

Another important observation is that the spheroids respond differently to the different scaffolds regarding the release of growth factors of interest for regenerative medicine. VEGF, FGF-2, and PDGF are mediators that act either as recruiters of endothelial cells to initiate angiogenesis or as inductors of proliferation, migration, or differentiation of progenitor cells [87]. Osteospheres interacting with scaffold surfaces after pretreatment with serum proteins released higher levels of these GFs and, in the case of PDGF, a factor already approved by the FDA for uses in regenerative medicine [88], the presence of a biomimetic coating increased such release. It can be suggested that apatite precipitation creates micro-environments capable of favoring cell-cell interaction that impacts on the production of some mediators by HOb spheroids. Considering that the local delivery of growth factors in damaged tissue is an important component of the bioengineering triad, the present results point that modifications on the scaffold surface such as biomimetic coatings may contribute to locally induce the production of at least some of these factors by cells from the material-tissue interface.

Nevertheless, other approach for the delivery of biological mediators of bone repair involves the direct use of the scaffolds as carriers. In the case of rhBMP-2, the development of novel biomimetic scaffolds becomes relevant as the FDA-approved collagen sponges are not suitable for the regeneration of larger defects. The idea of adding rhBMP-2 to the PLA-CaP scaffolds aims to increase the osteoinductive potential of the biomaterial, while rhBMP-2 have more affinity to apatite than biodegradable polymers [89,90]. In this sense, the present results shown that the 3D PLA-CaP scaffolds can effectively load rhBMP-2, according to the levels detected by ELISA, maintaining similar concentrations to those found on the absorbable collagen sponges (ACS) that comes in the INFUSE^®^ kit [90].

The data from the in vivo assessment demonstrated that all versions of the PLA-CaP investigated were biocompatible with rat calvaria, with the absence of inflammatory process. 3D PLA scaffolds promoted new bone formation after six months, even without presence of rhBMP-2, in agreement with previous reports showing that PLA supports bone formation [89]. However, the use of PLA-CaP-BMP2 scaffolds resulted in more bone formation than PLA-CaP and uncoated PLA scaffolds after six months of implantation.

In summary, this study demonstrated that PLA-CaP scaffolds could be produced as 3D-printed structures by the FDM technique, followed by a simple protocol of alkaline treatment and immersion in a biomimetic solution, with good reproducibility and accuracy, fundamental characteristics for graft manufacturing using personalized digital models (Computed tomography). Furthermore, after interaction with complex biological media, these scaffolds presented interesting biological responses, with increased cell migration or proliferation, production of differentiation markers, and release of growth factors, as observed in the assessment employing a novel 3D osteosphere in vitro model. Furthermore, the pre-clinical in vivo results showed considerable levels of newly formed bone in PLA-CaP scaffolds and even higher bone formation with the association of BMP2.

## 5. Conclusions

In this work, 3D-printed PLA scaffolds were successfully produced with pore size of 500 μm, 50% porosity, and mechanical properties similar to trabecular bone in a reproducible way. The biomimetic method was used to enhance scaffolds’ bioactivity by deposition of homogeneous and uniform apatite (CaP) layer on external and inner surfaces of 3D PLA scaffolds. The functionalization of 3D PLA scaffolds with a CaP coating (PLA-CaP) improved its properties in the presence of a complex biological media, inducing desirable responses from human osteoblasts (HOb). The association of 3D PLA-CaP with spheroids could be a new strategy for tissue engineering, since it could reduce the time of repair in situ by expression of biomolecules and growth factors. In vivo assays showed that PLA-CaP scaffolds plus rhBMP-2 produced biocompatible and bioactive scaffolds, with enhanced osteogenesis. In summary, using a scalable 3D printing technique and following simple protocols for surface biomimetic functionalization, it was possible to obtain scaffolds with good reproducibility and accuracy, fundamental characteristics for the manufacture of implantable biomaterials using customized digital models, besides presenting suitable surface properties to improve bone defect treatment. Moreover, the potential of these biomaterials as vehicles for local release of calcium and phosphate ions, growth factors and proteins in damaged tissues can be highlighted. The clinical relevance of these pre-clinical trials comes from the proposal of 3D printing technologies for manufacturing personalized scaffolds as alternatives for autologous grafts for long-term applications such as craniofacial reconstruction involving larger damaged areas.

## Figures and Tables

**Table 1:** Ionic concentration of blood plasma, 1.0 and 1.5 SBF solutions.

Solution	Concentrations (mol/m^3^)							
	Na^+^	K^+^	Mg^2+^	Ca^2+^	Cl^−^	HCO_3_^−^	HPO_4_^3−^	SO_4_^2−^
Blood plasma	142.0	5.0	1.5	2.5	103.0	27.0	1.0	0.5
SBF	142.0	5.0	1.5	2.5	147.8	4.2	1.0	0.5
1.5 SBF	213.0	7.5	2.3	3.8	221.7	6.3	1.5	0.8

**Table 2:** Mechanical properties and Archimedes calculus values (n= 5 ± SD). * Bose et al., 2013 [63].

	Uncoated PLA	PLA-CaP	Trabecular Bone *
Compressive strength (MPa)	20.50 ± 1.95	18.22 ± 2.67	2–20
Compressive Modulus (GPa)	0.512 ± 0.24	0.510 ± 0.11	0.1–2.0
Density (g/cm^3^)	1.23 ± 0.06	1.21 ± 0.02	–
Pore volume (mm^3^)	42.64 ± 6.49	41.61 ± 5.17	–
Porosity (%)	49.93 ± 5.28	49.09 ± 3.22	30–90
